# NMR in the Service of Wine Differentiation

**DOI:** 10.3390/foods10010120

**Published:** 2021-01-08

**Authors:** Marko Viskić, Luna Maslov Bandić, Ana-Marija Jagatić Korenika, Ana Jeromel

**Affiliations:** 1 Department of Chemistry, Faculty of Agriculture, University of Zagreb, Svetosimunska 25, 10 000 Zagreb, Croatia; mviskic@agr.hr; 2 Department of Viticulture and Enology, Faculty of Agriculture, University of Zagreb, Svetosimunska 25, 10 000 Zagreb, Croatia; amjagatic@agr.hr (A.-M.J.K.); amajdak@agr.hr (A.J.)

**Keywords:** wine, wine authenticity, geographical differentiation, NMR, SNIF-NMR, isotope analysis, irm-NMR

## Abstract

NMR is a swift and highly reproducible spectrometric technique that makes it possible to obtain spectra containing a lot of information about the sample analyzed. This approach helps major components be described in complex mixtures such as wine in just one analysis. Analysis of wine metabolites is very often used to understand the impact of geographical origin or variety on wine quality. NMR is often used for tracing the geographical origin of wine. Research on NMR metabolic effects of geographical origin is of great importance as the high added value of wines results from compliance with state legislation on the protected denomination of origin (PDO) and protected geographical indication (PGI) for the administration of the appellation of wines. A review of NMR with emphasis on SNIF-NMR in the analysis of wine authenticity is given. SNIF-NMR remains a method of choice for the detection of wine chaptalization as it is the only approach which provides position-specific information on the origin of sugar in wine. However, the sample preparation step, which lacks major improvements since its conception, is strenuous and expensive, and suffers from drawbacks in terms of low sample throughput. Mainstream 1D and 2D NMR experiments provide a fast and affordable way to authenticate wine based on the geographical origin, vintage, and variety discrimination, and include a simple and non-destructive sample preparation step. With this approach, spectral data processing often represents a crucial step of the analysis. With properly performed NMR experiments good to excellent differentiation of wines from different vintages, regions, and varieties was achieved recently.

## 1. Introduction

In the time of globalization, wine is truly one of the few products whose quality is primarily determined by its uniqueness and recognizability, based on grape varieties, origin, and technology. Practical wine production is no longer based on empiricism, whether they are mass-produced wines or small exclusive series of so-called boutique wines. A prerequisite for this is the rich scientific work and the connection of research in all scientific disciplines that can contribute to solving certain problems in practice. Knowledge of the chemical composition of grapes and wines is essential, and analytical chemistry is the best tool to achieve this. In the 19th century, analytical methods focused on the determination of major wine components such as ethanol, organic acids, and sugars. The development of chromatographic techniques in the early 1900s and particularly the development of gas chromatography in the early 1950s ushered in a new era of discovery for analytical chemists [1]. The components of wine flavor were revealed by gas chromatography. As modern analytical methods developed, so did knowledge of wine chemistry and biochemistry itself. The spectroscopic methods applied in the field of analysis of wine or grape include a broad range of techniques, covered by atomic spectroscopic methods such as atomic absorption spectroscopy (AAS) and inductively coupled plasma (ICP) and several molecular spectroscopic methods such as infrared- and ultraviolet/visible spectrophotometry, mass spectrometry (MS), and nuclear magnetic resonance (NMR) spectroscopy. NMR spectroscopy is based on the observation that certain atomic nuclei with a non-zero intrinsic nuclear magnetic moment, when placed in an external magnetic field, can be perturbed by oscillating radio-frequency field in near resonance conditions. Perturbed nuclei undergo free induction decay and emit an electromagnetic signal with a frequency characteristic of the chemical environment at the nucleus and its neighboring nuclei. Interaction between the magnetic moments of connected nuclei produces a coupling pattern in the signal, providing structural information about the analyte. The signal intensity, or its area, is proportional to the number of nuclei in resonance. These observations allowed for the use of NMR as one of the most advanced, sensitive, and reliable techniques used not only for the determination of molecular structure but also for quantitative analysis of real samples, purity determination, process control, medical imaging, and agricultural applications [2]. In the field of food analysis, NMR is used in compositional profiling, determination of authenticity, geographical origin of food components, and the effect of food storage and processing [3]. NMR represents the best nontargeted approach to food screening, as the majority of metabolites can be detected from a single spectrum with minimal sample destruction.

## 2. Application of NMR in Wine Analysis

### 2.1. Diversity of NMR Experiments in Wine Analysis

The application of NMR in wine science has evolved significantly in recent decades. NMR can be used to detect and quantify major compounds in wine: sugars, amino acids, lactic acid, acetic acid, etc., although other more available and recognized methods with somewhat better repeatability such as NIR, HPLC, and GC are usually applied. Nevertheless, the application of NMR has been developing in other fields of wine analysis. NMR was used to characterize the metabolome of intact grape berries [4] or berry extracts to estimate the maturity level before harvesting [5], and to assess the influence of terroir [6] and cultivation practices [7] on grape berry composition. Metabolic profiling of wines by NMR was used to group and compare wine samples [8,9,10]. NMR spectra of wines can show inherent genotypic variations among cultivars, which makes NMR a powerful tool for varietal classification of wines [11]. Nontargeted ^1^H NMR was used for recognition of Czech wine varieties, but with a very variable prediction outcome which ranged from 45% to 96% between cultivars [12]. Wine analysis by NMR can be nontargeted, as is the case of fingerprinting, which is not used for recognition of specific metabolites, involves recording multiple spectra under the same conditions, and relies heavily on multivariate data analysis to find patterns that could be used for sample discrimination. Another approach is targeted analysis, which enables the identification of selected key components of wine. The targeted approach commonly employs additional structural elucidation NMR experiments such as J-res, COSY, HMBC, HSQC, and TOCSY. In one targeted approach study, Godelmann et al. (2013) grouped wines by grape varieties and production practices by identification of shikimic acid as the marker of grape variety and caftaric acid as the marker of wine oxidation. In the same study, a nontargeted approach was used to recognize patterns in the whole spectral region and distinguish between 600 samples of German wines with an excellent prediction of over 95% [13]. Targeted and nontargeted approaches were used to investigate the influence of terroir on the composition of Italian wines made from Fiano di Avellino grapes by fermentation with commercial and autochthonous yeasts. By the targeted approach, wine discrimination was based on the tracking of six different metabolites [14]. Italian Lambrusco wines were analyzed by ^1^H NMR fingerprinting to observe the differences between varieties [15]. Imparato et al. have used ^1^H NMR to identify the varieties in wine blends, but with unsatisfactory precision [16]. Concentration changes of different compounds during fermentation can be monitored, both to expand understanding of the biochemistry of fermentation and to establish new procedures for winemaking quality control [17,18]. The effect of different yeast strains on the metabolic changes in monovarietal wine and their fermentative efficiency was studied by NMR with the aid of principal component analysis (PCA), partial least squares regression (PLS), and orthogonal partial least sqares regression (OPLS) at various fermentation stages [19]. Different wine aging conditions were monitored by methods for measurement of antioxidant activity and comparison of 1D and 2D NMR spectra of phenolic species in wines [20]. NMR was used to investigate changes induced by pathological processes in downy mildew and *Botrytis cinerea* infected grape and in differences in the composition of produced Champagne wine [21,22]. NMR has been used extensively to establish a link between the metabolome and the parameters influencing wine quality, such as grape variety [13,23,24], production practices [25,26], terroir [27], sensory profile [28,29], vintage [30], and aging [31]. Varietal and regional specificities of wines as well as their antioxidant profile can be better understood by identification of minor wine constituents by NMR [32,33,34], such as polyphenols [35,36] and stilbenes [37]. The versatility and complementarity of 2D NMR experiments were crucial for the determination of complex structures of anthocyanins in grape and wine [38,39]. Solid-state CP-MAS NMR was used to identify insoluble deposits in bottled red wines [40,41] and to investigate the interactions between yeast cell wall and anthocyanins during the fermentation process [42]. NMR was used to identify the compounds responsible for sensory properties of wine [43,44,45,46] or to understand their interaction with major wine components and macromolecules. Dufour and Bayonove have studied the capacity of catechins to bind aroma compounds by exponential dilution analysis and ^1^H NMR spectroscopy [47]. Structures of the adducts between varietal thiols and other aromatic compounds with major wine components were studied by 1D and 2D NMR methods to explain the wine aroma loss during storage [48,49,50]. Remy et al. have investigated the change of wine color by the reaction of anthocyanins and flavan-3-ols in model wine and confirmed the structures of the products by COSY, HSQC, and HMBC [51]. Oliveira et al. explored the behavior of vitisin B pigments and their acid-base properties in model wine solutions by ^1^H, ^13^C, HMBC, and HSQC [52]. The interaction of wine tannins with salivary proteins, a cause of astringency, was investigated by several methods including solid-state HR-MAS and 2D NMR techniques to track the chemical shifts of the peptides after the addition of tannins [53,54]. Diffusion-ordered spectroscopy (DOSY) was applied to track the evolution of polyphenols and other polymers generated during the storage of Port wines [55]. Wine spoilage in unopened bottles was confirmed by ^1^H and ^13^C NMR detection of acetic acid formed by ethanol oxidation. These methods required the use of a specially modified horizontal bore magnetic resonance imaging magnet and relied on a water suppression pulse sequence to trace ethanol, acetic acid, and acetaldehyde signals, but ensured complete preservation of the sample [56,57].

### 2.2. Some Features of NMR Wine Analysis and Data Handling

NMR has quickly gained popularity in wine analysis due to being a sample preparation step which is simpler and faster when compared to HPLC, GC-MS, or other instrumental techniques and classical methods. These approaches often require a purification and preconcentration step by LLE, SPE, or SPME and sometimes analyte derivatization. In contrast, when NMR is used, sample preparation involves wine pH adjusting with a suitable buffer, addition of D_2_O, an internal standard solution, and direct transfer of the sample to the NMR tube [9,12,13,14,16,27] or freeze-drying of wine prior to buffer addition [8,11,15,19,22]. If a sample consists of grape must, freeze-drying should be avoided as non-fermented sugar content would not allow proper elimination of water [17]. Simple additional steps such as centrifuging and degassing can be included [9,15]. In some reports [29,30], NMR spectra were recorded on wine samples with addition of D_2_O and no prior pH adjustment, which should be avoided due to possible peak shifts in the recorded spectra. When targeted analysis is performed, the influence of pH adjustment on the target metabolites should be taken into consideration. In one example, wine was evaporated to dryness and the residue was dissolved in D_2_O, which makes the method unsuitable for the determination of volatile metabolites [26]. Sample preparation for solid-state NMR includes isolation of target compounds and macromolecules by naturally occurring precipitation followed by precipitate freeze-drying [40,41]. Thanks to the improved sensitivity and resolution of modern NMR spectrometers, a large number of wine components are detected simultaneously, which results in highly complex spectra and significant peak overlaps. The latter can be somewhat evaded by the use of solvent suppression techniques, such as WET (water suppression enhanced through T_1_ effects), WATERGATE, and NOESY (Nuclear Overhauser effect NMR spectroscopy) presaturation sequences [58,59]. A serious drawback of NMR in food analysis is represented by the fact that tuning and resolution can be significantly altered between acquisitions, which may affect the intensity of all peaks in the spectra. Prior to data analysis, spectral pre-processing by baseline offset correction, phasing, peak alignment, and bucketing or binning has to be performed to reduce the acquired data size and minimize peak shift effects which arise from instrumental variations, pH dependence of the shift, and composition differences between wine samples [60]. Bucketing is performed by parsing spectra into small segments and creating a new spectrum containing smaller amounts of data. When conducted improperly, bucketing can result in a loss of information and incorrect bucket loads [61,62]. Various spectral alignment algorithms such as correlation optimized warping (COW), recursive segment-wise peak alignment (RSPA), interval correlation shifting (icoshift), fuzzy warping (FW), or the generalized fuzzy Hough transform (GFHT) can be used [63,64,65,66,67,68]. After the spectral processing, data are analyzed by a chemometric approach which usually reduces complex data to a smaller number of independent variables. The chemometric approach is used due to the high variability of the biological samples, including wine, and can clarify the initially inconclusive results, especially by combined analysis of data obtained by different techniques like ^1^H NMR, SNIF-NMR, and IRMS [69]. The most common unsupervised methods used for the extraction of useful information are principal component analysis (PCA) and hierarchical cluster analysis (HCA). Linear discriminant analysis (LDA), partial least-squares discriminant analysis (PLS-DA), K-means neighbors (KNN), soft-independent modeling class analogy (SIMCA), and artificial neural networks (ANN) belong to the group of supervised methods [70,71]. The main purposes of the chemometric methods are to describe sample variability, show sample clustering, and establish a possible pattern that could be used to recognize the authenticity of wines regarding the origin, variety, or some other factor [72,73]. Models built by both unsupervised and supervised methods must be carefully validated to avoid overfitting and false classification conclusions [74]. Commercial solutions like WineScreener™ facilitate the comparison of nontargeted spectral ^1^H NMR data with the reference spectra [75]. The major practical disadvantage of NMR in wine science is the unavailability of high-resolution NMR instruments in many enological and food analysis laboratories and the relatively high cost of their maintenance. Recently, handy “benchtop” NMR instruments, containing strong rare-earth magnets and operating without coolants, have appeared on the market, but which have lower working frequencies (up to 100 MHz, as of 2020) and can routinely be used to track major wine components. An overview of application of NMR in wine analysis is given in Table 1.

### 2.3. NMR as a Powerful Tool for Identification of the Geographical Origin

One of the first works dealing with NMR use in wine classification was by Vogels et al. (1993) who applied ^1^H and ^13^C nuclear magnetic resonance (NMR) spectroscopic methods in the differentiation of 53 German white wines from the regions Rheinhessen, Rheingau, and Mosel-Saar-Ruwer. The presented results demonstrated the possibilities of using both methods in combination with multivariate analysis techniques in the analysis and classification of German white wines [76]. A study published by Brescia et al. (2002) confirmed the possibility of geographical origin characterization of 41 Italian red wines from Apulia using ^1^H NMR spectrometric determination dividing them into three groups [8]. The same author [77] applied NMR in combination with high-performance ion chromatography and inductively coupled plasma emission spectroscopy on 33 wine samples coming from three Slovenian wine-growing regions and Apulia (Southern Italy). Again, good classification and prediction of the origin between Slovenian and Italian wines was achieved due to the amino acids isoleucine, proline, and citrulline; even though previous works pointed out possible differentiation of wine-growing regions when speaking about Apulia and Slovenia wines, separation was not satisfactory, probably due to the small differences in geoclimatic conditions between these regions. A combination of Fourier-transform–Near-infrared spectroscopy (FT-NIR) and ^1^H NMR spectroscopy was used to discriminate wines containing anthocyanins that originated from black rice and grapevine with the main purpose to detect adulteration of wine, with better results obtained on NMR spectra [78]. The first study dealing with Chinese wine classification and separation by use of ^1^H NMR spectroscopy and PCA was performed by Du et al. (2007), showing good separation among twenty-one bottled wines from three different areas (Shacheng, Changli, Yantai) while Fan et al. (2018) demonstrated that ^1^H NMR spectroscopy combined with multivariate analysis is an effective tool for verifying the authenticity of Chinese wines [23,79]. Application of ^1^H NMR metabolic data for wine analysis and authenticity determination of Chinese wines (Cabernet Sauvignon and Beihong) from two regions was also conducted by Gougeon et al. (2018) [80]. The method allowed the quantification of 33 metabolites including sugars, amino acids, organic acids, alcohols, and phenolic compounds that were successfully used for wine separation as a function of terroir and cultivar. The same author investigated red wines from the main French DPO (Bordeaux, Beaujolais, Burgundy, Côtes du Rhône, Languedoc-Roussillon, and Loire Valley) and different vintages (2004–2017) by qNMR spectroscopy coupled to multivariate data analysis [81]. Successful separation of Bordeaux wines, as well as wines from two sub-regions (Liburnais and Medoc), was made based on forty quantified compounds. The same result but with different grape varieties (Cabernet Sauvignon, Campbell Early, Shiraz, Merlot) from France, USA, Korea, and Australia was achieved by ^1^H NMR spectroscopy and PCA and PLS. The metabolites contributing to the separation were assigned to be butane-2,3-diol, lactate, acetate, proline, succinate, malate, glycerol, tartarate, glucose, and phenolic compounds. In addition, good separation of Cabernet Sauvignon grapes but from Australia, France, and the USA was defined primarily based on different proline concentrations [82]. In Italy, characterization of 20 Aglianico wines from different areas of the Basilicata region was performed by the use of ^1^H NMR and ^13^C NMR spectroscopy and the results confirmed the important role of succinic acid, proline, 2, 3-butanediol, and glycerol in differentiation according to their geographical origin [83]. Sixteen Aglianico wines from three different vineyards were analyzed by ^1^H NMR and have been successfully differentiated by six metabolites (hydroxyisobutyrate, lactic acid, succinic acid, glycerol, fructose, D-glucuronic acid). The findings of this work indicated NMR spectroscopy as a rapid and objectively sound technique for geographical wine quality identification [14]. Papotti et al. (2013) also demonstrated the ability of ^1^H NMR spectroscopy, coupled with multivariate data analysis methods, to be a useful model in the classification of 110 protected denomination of origin (PDO) Lambrusco wines of Modena according to the varietal source. Good discrimination was found by analyzing the low-frequency spectral region using PLS-DA revealing butane-2,3-diol, lactic and succinic acids, threonine, and malic acid to be important compounds for varietal discrimination [15]. In the same period, a group of Greek researchers, Anastasiadi et al. (2009), used ^1^H NMR metabolomics for the discrimination of 67 Greek wines from the Agiurgitiko, Mandilaria, Moschofilero, and Asyrtiko varieties grown in the Nemea and Santorini regions. Classification of wines according to the same variety in different production years as well as different wines in the same wine growing region was performed based on their phenolic extract obtained by polymeric adsorbent XAD^®^ resin and monitored by NMR in combination with multivariate analysis [24].

## 3. SNIF-NMR

Due to the increasing mass production and consumption of wine, adulteration poses a serious financial problem. The complexity of food matrices and the similarity of analytes in foodstuff make adulteration detection a continuously challenging task. G. Martin and M. Martin from the University of Nantes—CNRS and Eurofins inc. in France developed site-specific isotopic fractionation-nuclear magnetic resonance (SNIF-NMR), also called isotopic ratio measurement by NMR (irm-NMR) in the recent literature [84,85], in the 1980s as a method for detecting chaptalization, or deliberate addition of sugar to the grape must before fermentation [86,87]. Earlier chaptalization monitoring was based on the detection of impurities originating from the sugar production process, but with technical improvements in sugar refining, these methods were deemed insufficient. Application of stable isotope techniques was suggested, as it was already recognized in other fields of research such as geochemistry and biochemistry. Variations in isotope ratios can be accurately detected by mass spectrometry (IRMS) and SNIF-NMR. While IRMS can determine only a mean value of the deuterium content of a given chemical species, SNIF-NMR contains the structural information inherent to NMR methods, thus allowing localized determination of isotopic ratios at individual sites of an analyte [88].

### 3.1. Features of Deuterium Nuclei and Deuterium NMR

The most exploited nucleus in NMR spectroscopy is ^1^H, due to large natural abundance, high sensitivity, and narrow chemical shift. On the other hand, observation of the ^2^H nuclei resonances is strenuous due to their low natural abundance (0.015%) and a smaller gyromagnetic ratio of 4.1064 × 10^7^ rad T^−1^ s^−1^, or one-sixth of the value for ^1^H nuclei. Due to the low gyromagnetic ratio of the ^2^H nucleus, the excess spin population of nuclei is reduced and the sensitivity of ^2^H NMR detection is largely diminished, which in turn resolves lower. Although its nuclear spin value is an integer, deuterium nuclei have a small quadrupole moment and can still produce spectra with high resolution and acceptable sensitivity. Its relaxation is dominated by the quadrupolar mechanism and intensity enhancement from the nuclear Overhauser effect is negligible. Deuterium–deuterium couplings are usually not observed due to the low abundance, while small scalar couplings between deuterium and proton are removed by acquiring ^2^H spectra with a ^1^H decoupling mode to simplify the spectra. Field frequency locking is mostly performed on ^19^F, although an improved approach with an external unit ^1^H lock is also used [89,90]. Since quantification has to be performed with accuracy, the repetition time has to have at least quintuple duration of T_1_ relaxation time of deuterium, and a satisfactory S/N ratio is obtained by precise determination of the 90° pulse duration. Longer experiment times and larger samples due to the low abundance of deuterium have been significantly reduced by advances in magnet technology, probe design, the introduction of cryoprobes, and pulsed-field gradients [84,91]. Deuterium accounts only for about 0.01% of all the hydrogen atoms in the sample. Due to the low natural abundance, if a molecule contains a ^2^H nucleus, it is unlikely that a second deuterium atom will be present in it, and only monodeuterated molecules will be present in the sample. Thus, each ^2^H NMR signal will belong to one monodeuterated isotopomer [92].

### 3.2. The Principle of SNIF-NMR for Food Authentication

**Figure 1:**
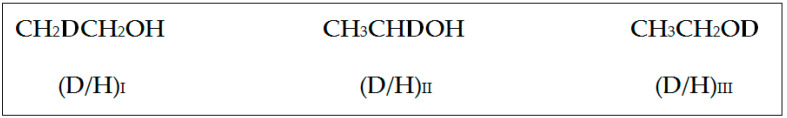
Isotopomers of ethanol detected by ^2^H NMR spectroscopy and the respective isotopic ratios.

**Figure 2:**
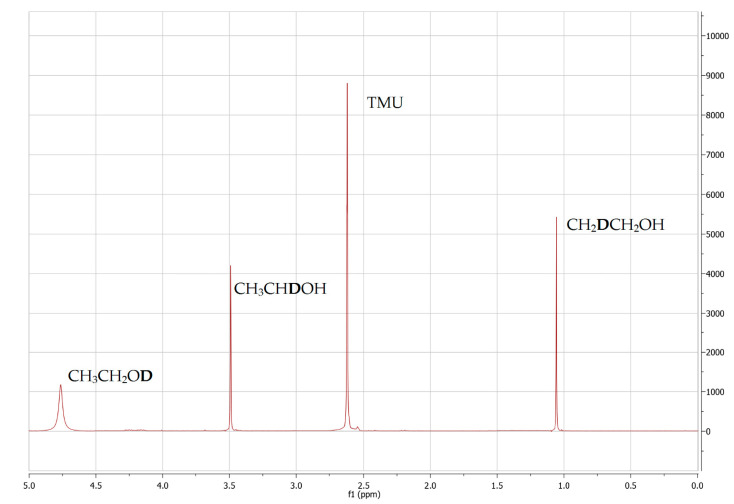
Spectrum ^2^H-NMR.

The principle of wine SNIF-NMR lies in the comparison of the ratios of the signals from methyl and methylene sites in the ^2^H NMR spectrum of ethanol [93]. A major component of wine, ethanol is the most suitable NMR probe for chaptalization tracking, as it is produced from natural or added sugar during fermentation. Since accurate quantification is of utmost importance in SNIF-NMR, due to the low abundance of deuterium, SNIF-NMR is never performed directly on wine samples, but always on a target analyte which has to be separated and purified from wine. It is easier to isolate ethanol from wine by distillation and to assign its spectra in comparison to carbohydrates [94]. However, precaution is needed during the sample preparation steps to avoid isotopic fractionation artifacts—the presence of residual sugars at the end of fermentation has to be checked, the distillation of ethanol has to be quantitative, and its evaporation before or during the NMR measurement has to be avoided [95,96]. Another fact which made SNIF-NMR applicable for chaptalization detection is the observation that deuterium atoms in the methyl and methylene site in ethanol mostly originate from different sources. Distribution of deuterium in sugar is a function of different photosynthetic pathways (C_3_, C_4_, CAM), the environmental conditions of harvesting, and the deuterium abundance in water used by the plant, which shows geographic variations [97]. The disadvantage of deuterium’s low abundance is exploited as an advantage in SNIF-NMR, which is based on deuterium labeling by nature on the specific molecular sites. Natural isotopic fractionation is a result of physiological transformations in living organisms, which lead to a non-statistical distribution of deuterium and other isotopes in compounds of natural origin [98]. By SNIF-NMR, the hydrogen isotope ratio is determined for the methyl group of ethanol (D/H)_I_, which is related to sugar and its origin, and for the methylene group of ethanol (D/H)_II_, which originates mostly from water and can give an insight into the climatic and regional origins of the produced wine (Figure 1 and Figure 2). The ratios (D/H)_I_ and (D/H)_II_ can be determined from NMR spectra by the following equations:

(1) $${\left(D/H\right)}_{I}=1.5866\;T_{I}\frac{m_{\mathrm{st}}}{m_{a}}\frac{{\left(D/H\right)}_{\mathrm{st}}}{t_{m}^{D}}$$

(2) $${\left(D/H\right)}_{\mathrm{II}}=2.3799\;T_{\mathrm{II}}\frac{m_{\mathrm{st}}}{m_{a}}\frac{{\left(D/H\right)}_{\mathrm{st}}}{t_{m}^{D}}$$

where (D/H)_I_ is the isotope ratio at the methyl site, (D/H)_II_ is the isotope ratio at the methylene site, T_I_ and T_II_ are ratios of the areas of the ^2^H NMR signals at the specific molecular sites, m_st_ is the weight of the standard, m_a_ is the weight of the ethyl alcohol sample, $t_{m}^{D}$ is the volume ratio of ethanol within the sample, and (D/H)_st_ is the known isotope ratio of internal standard (TMU) [99]. The influence of various factors such as the yeast strain, fermentation parameters, and the concentration of endogenous sugar and nutrients on the deuterium isotopic ratio was checked. If the content of above 70% sugar is fermented into alcohol, these factors did not affect the (D/H)_I_ values in ethanol, while the (D/H)_II_ ratio can vary significantly. This allowed for the (D/H)_I_ ratio as a firm indicator of wine chaptalization [100,101,102,103]. The isotopic ratio (D/H)_III_ is not used in chaptalization detection due to the exchangeability of hydrogens at the hydroxylic group of the ethanol molecule. The isotopic abundances at the two relevant sites of ethanol are expressed as an absolute ratio, and calibration is performed against *N, N’*-tetramethylurea (TMU), or some other standard with a known isotopic composition [104]. In more recent works, the use of internal standard has been substituted by an electronically generated signal, which reduces the sample preparation time and the experiment duration, and improves S/N ratio [89,105,106]. A serious drawback of SNIF-NMR is a need for a distillation apparatus such as a Cadiot column and a larger amount of sample needed, while IRMS relies on the combustion of as little as 1 mg of the sample in monitored conditions [107]. SNIF-NMR provides the unmatched possibility for wine authentication with simple data interpretation. Yet, the sample preparation step, which was barely modified since the conception of SNIF-NMR, remains critical, as it is overly sensitive to external interferences such as incomplete distillation or unwanted isotope fractionation, and it has been described as tedious and expensive [108]. Combined with the long duration of the ^2^H NMR experiment, this approach results in a low sample throughput of a couple of samples per day.

The official method of chaptalization detection includes determination of (D/H)_I_ and (D/H)_II_ ratios in ethanol by SNIF-NMR and determination of (^13^C/^12^C) and (^18^O/^16^O) ratios by IRMS [104,109]. The carbon isotope signature is useful as the ^13^C value will increase if sugars from a C_4_ plant are added to the must. Deuterium NMR suffers from some limitations, such as a lack of resolution from overlapping signals and limited dynamic ranges due to quadrupolar relaxation, which limits the technique to analytes of lower molecular weight. In the last decade, there was increased interest in the exploration of the metabolically driven partition of ^13^C isotope by SNIF-NMR, as ^13^C nuclei have higher sensitivity owed to a larger natural abundance (1.108%) and gyromagnetic ratio (6.7263 × 10^7^ rad T^−1^ s^−1^), and a larger chemical shift range than deuterium [84,110,111,112,113]. The natural abundance of ^13^C is approximately 100 times higher than deuterium, but quantification of ^13^C by NMR can be complicated due to the nuclear Overhauser effect and a very narrow range of ^13^C/^12^C ratios found in organic molecules. Additionally, longitudinal relaxation times are very long, which leads to extended acquisition [114,115]. With the improvements in pulse techniques, suppression of the nuclear Overhauser effect was achieved by inverse gated decoupling [116], and an increase in sensitivity was observed by the use of polarization transfer from sensitive ^1^H to less sensitive ^13^C nucleus [111]. Site-specific ^13^C NMR of wine did not provide significant improvement in discrimination between added sugars from C_3_ and C_4_ plants [117] but can be exploited in wine authentication since *δ*^13^C (a relative difference of isotope ratios) of the methylene group in ethanol can be used as a marker of grape growing climatic conditions [118]. In addition, ^13^C SNIF-NMR was used for the detection of corn syrup adulteration in products from CAM plants, such as pineapple juice [110].

### 3.3. Application of SNIF-NMR in Food Analysis

Since its conception and commercialization, various food and agricultural products were authenticated by stable isotope ratio analysis. SNIF-NMR was applied to discriminate vinegar made by synthetic acetic acid dilution from genuine vinegar made by fermentation. Origin identification is performed by SNIF-NMR determination of the (D/H)_CH3_ isotope ratio in acetic acid, sometimes in combination with ^13^C-IRMS [119,120,121]. Additional determination of ^18^O/^16^O ratio of water in wine vinegar by IRMS allows differentiation of wine vinegar made from raisins [109]. The connection between the ^2^H content in sugar and ethanol was applied to the detection of adulteration in other sweet food, such as fruit juices [122,123]. Whole fruits or fruit juices were also analyzed by SNIF-NMR to track their geographical origin [124,125]. SNIF-NMR was applied in the detection of honey adulteration by the addition of beet or cane sugar syrup or mislabeling of the botanical origin of honey. However, SNIF-NMR proved to be less sensitive than IRMS in the detection of sugar addition, as the minimum LOD of sugar spike was around 20%, which is not satisfactory. Additionally, some authentic sources of honey were not discriminated [126,127,128]. A few reports on sugar source detection by SNIF-NMR in maple syrup [129] and whey [130] were published. SNIF-NMR was used to discriminate natural versus synthetic origins of aromas, such as vanilla extract [131,132], and study the geographical origin of edible oils, such as salmon oil [88] and olive oil [133]. As the origin of sugar has tobe determined, in the case of wine, honey, syrups, and fruit juices, the sample preparation step will always involve sugar fermentation and distillation of formed ethanol. Fatty food analysis by SNIF-NMR focuses on the distribution of deuterium in lipid molecules, and for that purpose, the site-specific isotope ratios of the methyl esters of lipid extracts or free fatty acids are determined [88,133]. ^2^H/^1^H ratios determined by SNIF-NMR were used to distinguish between geographic origins of heroin and cocaine [134], while site-specific ^13^C/^12^C ratios determined by NMR and IRMS were used to distinguish between different sources of ascorbic acid [135].

## 4. SNIF-NMR in Wine Authenticity

### 4.1. Detection of Wine Chaptalization

The most common adulteration practice detected by SNIF-NMR is chaptalization. Sugar present in grapevine is produced by a C_3_ plant, while sugar and syrups from sugar cane and corn are derived from C_4_ plants with a distinct isotopic signature. Chaptalization with sugar from beetroot or another C_3_ plant can still be observed, as the methyl site isotopic ratio is significantly lower in ethanol produced by fermentation of beetroot sugar when compared to ethanol derived from grapevine sugar [136,137]. The degree of chaptalization by beetroot sugar can be expressed from the following equation:

(3) $$\%=100\frac{{\left(D/H\right)}_{I}^{S}-{\left(D/H\right)}_{I}^{\mathrm{COP}}}{{\left(D/H\right)}_{I}^{\mathrm{BEET}}-{\left(D/H\right)}_{I}^{\mathrm{COP}}}$$

(4) $$\%=100\frac{\delta^{13}C^{S}-\delta^{13}C^{\mathrm{COP}}}{\delta^{13}C^{\mathrm{CANE}}-\delta^{13}C^{\mathrm{COP}}}$$

where (D/H)_I,s_ is the isotopic ratio of the sample after normalization for the deuterium content of water [138], (D/H)_I,BEET_ is the mean value for the beet sugar reference (92.7 ppm), while (D/H)_I,COP_ refers to the cutoff value of the ratio for the geographic origin of the specific sample [104]. Experimentally determined isotopic ratios are compared to the official national and international wine databanks when authentication and geographical grouping are performed [139,140]. Generally, when the value of (D/H)_I_ for wine samples is lower than the value for grape sugar (101.8 ppm), adulteration with beetroot sugar is present. Conversely, when cane sugar, with a notably higher (D/H)_I_ value (112.0 ppm), is added, an increase in (D/H)_I_ ratio of ethanol will be measured. In some cases, chaptalization is performed by adding a mixture of cane and beet sugar and thus becomes more tedious to detect. However, ^13^C-IRMS would still be able to identify this kind of practice [104]. As a relatively easily accessible SNIF-NMR probe, ethanol remains the major wine component used for the detection of wine adulteration. Limited tests of adulteration of wine with other compounds were performed. Hermann has developed a method for tracking glycerol wine spiking by determination of three ^2^H/^1^H ratios in glycerol molecules. Isotopic ratios varied between glycerol formed in wines during fermentation, glycerol of lipid origin, and the one of synthetic origin, but only with a modest correlation [141]. In addition to site-specific deuterium ratios, ^13^C/^12^C and ^18^O/^16^O ratios can indicate adulteration by watering or ethanol addition [142,143]. Perini and Camin have measured the ^18^O/^16^O ratio of ethanol from 69 samples of Italian wines by IRMS. This approach was applied to detect watering down, as in adulterated wines, a relation between the *δ*^18^O of ethanol and the *δ*^18^O of the fermentation medium will not be linear [142]. In wine authenticity studies, the ratios of ^2^H/^1^H and ^13^C/^12^C in ethanol carry a signature about the grape variety, geographical provenance of wine and grape, and viticultural practices, and can be used in wine classification and authenticity testing [137,139]. Isotopic ratios of wine ethanol are usually not altered by winemaking practices, but there are some notable exemptions, such as must concentration by high-vacuum evaporation, which has to be considered during wine screening [144,145]. Passito wines produced by post harvesting drying of grape berries had a decreased value of *δ*^18^O of wine ethanol and water, while berry over-ripening on the plant resulted in an increase in *δ*^18^O signatures [146]. On the other hand, fermentation stopping, usually employed in the production of Italian Moscato wines, did not influence the (D/H)_I_ ratio, while a strong correlation between the (D/H)_II_ value and the stage of fermentation stopping was found [103].

### 4.2. SNIF-NMR in Wine Authentication

The beginning of stable isotope analysis in food science goes back to the past with the primary purpose of tracing the origin of a product and detecting a different kind of food adulteration. In the 1990s, the first methods based on stable isotope analyses of foods have been developed and acknowledged as official methods in the European Union (EU regulations 2670/90, 2347/91, and 2348/91). A review paper by Rossmann (2001) pointed out that routine application of stable isotope analysis is most advanced in the field of wine quality control because since 1990, the European Union (EU) established an official wine database for stable isotope parameters [147]. The geographic and climatic parameters, which influence the vine growing, can be correlated with the site-specific isotopic parameters, which thus become a reliable criterion for wine authentication. Many studies have focused on the influence of the geographic origin on the values of isotope ratios [148,149,150] and the resulting discrimination of wines by these differences with respect to the location of production [69,151,152,153,154,155,156,157,158,159]. Table 2 presents the use of SNIF-NMR in the detection of wine adulteration, investigation of geographical origin of wines, and wine production practices. One of the first reports on wine discrimination by provenance was published by Martin et al. who applied PCA based on measured isotope ratios and noted clustering of wines from three French regions thanks to different climatic features. If samples were selected within a single variety, better discrimination was achieved [137]. Camin et al. studied the differences among 4000 samples of Italian wines by combining isotopic ratios and geographic and climatic descriptors. Chemometric tools were used with data visualization maps to clarify large datasets. They concluded that *δ*^18^O and (D/H)_I_ are the two isotopic parameters most influenced by climatic and geographic conditions [150]. Since the geographic classification of wines depends on a multitude of factors, isotope ratio data should never be considered separately, but rather in combination with additional measured values and descriptive factors. When wines are discriminated by variety, it should be noted that the differences in isotope ratios might rather be caused by the climatic variations during grape growth, due to different responses in photosynthetic and maturation cycles between different varieties in years with slightly altered climatic conditions [98].

Stable isotope detection methods used for the geographical discrimination of wine rely on the analysis of only the major wine components, ethanol and water. The isotope ratios of grape water depend on the climatic conditions and harvesting period. This fact makes geographical discrimination and distinguishing between different appellations [162], based on the (D/H)_II_ ratio among other values, less robust than chaptalization detection. These methods require reliable and up-to-date databases [161]. When SNIF-NMR is used to trace the geographical origin of wines, it can be beneficial to combine climate-dependent isotopic data with the results of analyses of other species. In addition to performing SNIF-NMR, Day et al. have combined the determined isotopic ratios with the concentration of trace elements and micronutrients in soil determined by AAS. This model was used to distinguish between distant and near-production areas in France, and by applying LDA, resulted in a very good grouping efficiency [139]. Martin et al. (1996) reported an inter-laboratory comparison study by deuterium NMR spectrometry to define the repeatability (r) and the reproducibility (R) of the site-specific isotope ratio determinations in ethanol resulting from the fermentation of fruit juices. Fifteen laboratories used the same experimental protocol under different conditions (operator, spectrometer, a period) to ferment the fruit juices, distill the ethanol, and record the ^2^H NMR spectra [165]. The results achieved were considered to be acceptable by specialists. In that period, a group of Slovenian researchers published several works showing the discriminating potential of NMR methods reflecting the differences in Sauvignon and White Pinot wine composition from the Drava and Coastal regions [166], proceeding with further authenticity determination and geographical origin of 50 wines selected from the Coastal, Sava, and Drava regions of Slovenia [151]. Their work was expanded with the identification of amino acids in wines by one- and two-dimensional NMR and determination of minor compounds. In conclusion, they showed that high-field NMR can be a promising method for the non-destructive analysis of low molecular mass compounds in wine. Complete assignment of proton and carbon chemical shifts of 17 amino acids and of *γ*-aminobutyric acid commonly present in wines have been achieved in Sauvignon wine samples, while in 10 selected white wine samples (Chardonnay, Welsch Riesling, Sauvignon, and Rhine Riesling) from three wine-growing regions in Slovenia, the capacity of 1D and 2D ^1^H and ^13^C homo- and heteronuclear NMR were examined for the characterization of amino acids and anthocyanins [58,167]. Regional origin of monovarietal *Vitis vinifera* L. cvs. Bobal, Tempranillo, and Monastrell wines of Valencia have been also investigated by ^2^H NMR determination of the site-specific (^2^H/^1^H) ratios of fermentative ethanol by Giménez-Miralles et al. (1999). The results achieved pointed out reasonable differentiation at a micro-regional scale in terms of geographic provenance and grapevine genotypic features, even though microclimatic complexity and annual environmental variation of viticultural areas may influence regional origin discrimination from year to year [148]. The geographical origin of authentic Swiss wine samples (Pinot noir, Müller–Thurgau, Chasselas, Merlot, Silvaner, Gamay, Escala, Petite Arvine, Pinot Blanc, Dole) from the years 2000 (68 samples) and 2001 (82 samples) together with 12 commercial wines from the year 2000 was determined by^2^H/^1^H NMR and the results in combination with isotopic ratio mass spectrometry (IRMS), inductively coupled plasma–optical emission spectroscopy (ICP-OES), inductively coupled plasma–mass spectrometry (ICP-MS), and Fourier-transform–infrared spectroscopy (FT-IR) data were used to distinguish four main production zones of Switzerland [168]. In a recent work, Durante et al. determined the variations of isotopic ratios for (D/H)_I_, (D/H)_II_ by SNIF-NMR, together with *δ*^13^C, *δ*^15^N, and *δ*^18^O values and ^87^Sr/^86^Sr ratio in Lambrusco and Trentodoc wines grown on various soils and made by different production practices. The isotopic ratio (D/H)_I_ did not vary significantly, but a correlation with (D/H)_II_ and the fermentation conditions was noticed [159]. Kokkinofta et al. applied SNIF-NMR on 76 samples of Cypriot wines to distinguish between Xynisteri, Maratheftiko, Cabernet Sauvignon, and Shiraz varieties, their geographic origin, and vintage. PCA and OPLS-DA were applied to cluster the samples, and differentiation was significantly improved when additional results of macronutrients analysis were included in the models. The value of the isotope ratio (D/H)_II_ had a pronounced influence on vintage and vineyard effect discrimination [155]. Further improvement of data analysis for wine discrimination can be achieved by simultaneous chemometric analysis of isotopic ratio data together with results by other methods. In a work by Monakhova et al. (2014), the models for the prediction of grape variety, vintage and geographical origin were improved when data from ^1^H NMR fingerprinting were joined to SNIF-NMR and IRMS results [69]. In another report, Romanian wines were grouped by variety and vintage when the results from stable isotope measurements were processed together with 1D NMR fingerprints [163].

## 5. Conclusions

NMR is a powerful analytical tool in terms of simple, non-invasive, uncomplicated sample preparation techniques for both targeted and non-targeted analysis. It is widely used in wine analysis for studying metabolomics and tracing the geographical origin and authenticity of wine. In addition to a simple sample preparation, improvements in spectral data processing and pulse techniques have made NMR a method of choice for the investigation of different growing practices on the properties of wine, discovery of trace compounds, and monitoring of wine spoilage. Besides the excellent performance of NMR techniques, it is still capital equipment not so often available in most common laboratories for food analysis. In the field of food authentication analysis by isotope tracing, IRMS provides a quicker and more sensitive method, as the preparation of samples for SNIF-NMR in authentication studies is much more time consuming and requires special precautions. Even when the majority of isotopic distribution data for adulteration control is measured by SNIF-NMR, IRMS will still be required for determination of oxygen isotope ratios. Recent technical advances in NMR spectrometer coils and pulse techniques have somewhat increased sample throughput, but the laborious sample preparation step remains the main obstacle to wider use of SNIF-NMR, and the use of IRMS for adulteration studies is preferred. Nevertheless, a wide choice of NMR experiments have become increasingly accepted in applications of differentiation of wine as an essential goal of the vine-growing sector worldwide, especially in the EU.

## Figures and Tables

**Table 1:** Selected examples of application of nuclear magnetic resonance in wine analysis with emphasis on the variety of applications.

Discriminative Factor Investigated by NMR Metabolomics/Aim of NMR Study	Chemometric Method	NMR Technique	Reference
Grape berries; variety, harvesting time	PCA, PLS, OPLS	^1^H, J-res, COSY, HMBC, HSQC	[4]
Grape berries; variety, terroir	ANOVA, PCA	^1^H	[5]
Grape berries; cultivation practices	PCA	^1^H	[7]
Grape pulp; terroir, vintage	PCA, ANOVA, PLS-DA	HR-MAS, COSY, TOCSY, J-res, HSQC, HMBC	[6]
Geographical origin	PCA, HCA, DA	^1^H	[8]
	PCA, PLS-DA	^1^H, ^13^C, HMBC, HSQC	[15]
Terroir	PCA, ECVA	^1^H	[9]
	PCA, DA, HCA	^1^H, COSY, TOCSY, HSQC	[14]
Variety	PCA, PLS, HCA	^1^H, COSY	[11]
	PCA, PLS, HCA, ANOVA, RF	^1^H, ^13^C, J-res, COSY, HSQC	[12]
	PCA, LDA	^1^H	[23]
Cultivation technique	PCA, OPLS-DA	^1^H	[26]
	PCA, PLS	^1^H, ^13^C, COSY, HSQC, HMBC	[25]
Variety, geographical origin, and vintage	PCA, PLS-DA	^1^H	[24]
	PCA, LDA, MANOVA, MC	^1^H	[13]
Yeast strain applied in fermentation	PCA, HCA, DA, ANOVA	^1^H, COSY, TOCSY, HSQC, HMBC	[27]
Fermentation monitoring	-	^1^H	[17]
	PCA	^1^H	[18]
	PCA, PLS-DA, OPLS-DA	^1^H, TOCSY, HMBC, HSQC	[19]
Infection by plant pathogen	PCA, PLS-DA, HCA	^1^H, J-res, COSY, HMBC, HSQC	[21]
	PCA, OPLS-DA	^1^H, TOCSY, COSY, HMBC, HSQC	[22]
Sensoric profile	ANOVA, PLS	^1^H, COSY, HSQC	[28]
Sensoric profile, variety, cultivation practices	PCA, PLS-DA	^1^H	[29]
Vintage, ageing	PCA, PLS-DA	^1^H, TOCSY, HSQC	[30]
Ageing	-	^1^H, COSY, TOCSY, HSQC, DOSY	[55]
	PCA, PLS-DA, ANOVA	^1^H, TOCSY, HSQC	[31]
Wine blending	LDA, ANN	^1^H	[16]
Assessment of wine antioxidative potential and influence of aging conditions	-	^1^H, ^13^C, COSY, HSQC, HMBC	[20]
Identification and characterization of minor wine components with sensoric or bioactive role		Always a combination of some of the following techniques: ^1^H, ^13^C, COSY, NOESY, TOCSY, ROESY, HMBC, HSQC, HMQC	[32,33,34,35,36,37,38,39,43,44,45,46,52]
Interactions between wine components with sensoric role		CP-MAS NMR	[42]
		^1^H	[47]
		^1^H, ^13^C, HMQC, HMBC, COSY	[48]

**Table 2:** Use of SNIF-NMR in the detection of wine adulteration, investigation of geographical origin of wines, and wine production practices.

Purpose	Samples and Varieties	Country	Reference
Adulteration detection	354 samples; multiple varieties	Italy	[160]
Geographical discrimination, adulteration detection-watering	30 samples; multiple varieties	Italy, Germany, France	[161]
Adulteration detection-glycerol	-	Germany	[141]
Adulteration detection-sugar, Geographical discrimination	50 samples; 17 varieties	Slovenia	[151]
Geographical discrimination, Adulteration detection	5 varieties; Feteasca Regala, Feteasca Alba, Merlot, Cabernet Sauvignon	Romania	[156]
Adulteration detection-watering	69 samples; multiple varieties	Italy	[142]
Influence of must concentration on isotope ratios	42 must samples	France	[145]
Influence of fermentation stopping on isotope ratios	126 must samples, 18 commercial wines	Italy	[103]
Influence of grape withering on the isotope ratios	78 samples; Passito wines	Italy	[146]
Geographical discrimination	1383 samples; 14 varieties	Germany	[69]
	33 samples, 17 varieties	Slovenia, southern Italy	[77]
	166 samples; multiple varieties	France	[139]
	96 samples; Bobal, Tempranillo, Monastrell	Spain	[148]
	5220 samples; multiple varieties	Italy	[149]
	3948 samples; multiple varieties	Italy	[150]
	1496 samples; multiple varieties	Italy	[152]
	100 samples; Merlot, Cabernet Sauvignon, Riesling, Chardonnay, and Italian Riesling	China	[153]
	76 samples; Xynisteri, Maratheftiko, Cabernet Sauvignon, and Shiraz	Cyprus	[154]
	102 samples; multiple varieties	Slovenia	[155]
	54 samples; Nebbiolo, barbera	Northern Italy	[157]
	78 samples; Mourvedre, Rolle, and Syrah	Lebanon	[158]
	206 samples; Lambrusco, Trentodoc	Italy	[159]
	259 samples; multiple varieties	France	[162]
	56 samples; Cabernet Sauvignon, Merlot, Feteasca Neagra, Pinot Noir, and Mamaia	Romania	[163]
	445 samples; multiple varieties	Italy	[164]

## Data Availability

The data presented in this study are available on request from the corresponding author. The data are not publicly available due to privacy concerns.
